# Circulating Angiopoietin-2 Is a Marker for Early Cardiovascular Disease in Children on Chronic Dialysis

**DOI:** 10.1371/journal.pone.0056273

**Published:** 2013-02-08

**Authors:** Rukshana C. Shroff, Karen L. Price, Maria Kolatsi-Joannou, Alexandra F. Todd, David Wells, John Deanfield, Richard J. Johnson, Lesley Rees, Adrian S. Woolf, David A. Long

**Affiliations:** 1 Nephro-Urology Unit, UCL Institute of Child Health and Great Ormond Street Hospital for Children NHS Trust, London, United Kingdom; 2 Department of Chemical Pathology, Great Ormond Street Hospital for Children NHS Trust, London, United Kingdom; 3 National Centre for Cardiovascular Disease Prevention and Outcomes, University College London, London, United Kingdom; 4 Division of Renal Diseases and Hypertension, University of Colorado, Denver, Colorado, United States of America; 5 Institute of Human Development, University of Manchester and the Royal Manchester Children’s Hospital, Manchester, United Kingdom; Beth Israel Deaconess Medical Center, United States of America

## Abstract

Cardiovascular disease (CVD) is increasingly recognised as a complication of childhood chronic kidney disease (CKD) even in the absence of diabetes and hypertension. We hypothesized that an alteration in angiopoietin-1 and -2, growth factors which regulate endothelial and vascular function could be involved. We report that the endothelial survival factor, angiopoietin-1 is low in children with pre-dialysis CKD whereas the pro-inflammatory angiopoietin-2 is elevated in children on dialysis. In dialysis patients, angiopoietin-2 positively correlated with time on dialysis, systolic blood pressure, and carotid artery intima media thickness. Elevated angiopoietin-2 levels in dialysis versus pre-dialysis CKD patients were also associated with an anti-angiogenic (high soluble VEGFR-1 and low VEGF-A) and pro-inflammatory (high urate, E-selectin, P-selectin and VCAM-1) milieu. Ang-2 was immunodetected in arterial biopsy samples whilst the expression of VEGF-A was significantly downregulated in dialysis patients. Serum urate correlated with angiopoietin-2 levels in dialysis patients and addition of uric acid was able to induce rapid release of angiopoietin-2 from cultured endothelial cells. Thus, angiopoietin-2 is a marker for cardiovascular disease in children on chronic dialysis and may act as an anti-angiogenic and pro-inflammatory effector in this context. The possibility that the release of angiopoietin-2 from endothelia is mediated by urate should be explored further.

## Introduction

Children with chronic kidney disease (CKD) develop early onset cardiovascular disease (CVD). [1] Manifestations of CVD in childhood CKD include arterial stiffening [2] and calcification, [3] premature atherosclerosis, [4] and left ventricular hypertrophy. [5] Over time, CKD developing in children is associated with increased cardiovascular mortality that markedly accelerates once dialysis is initiated. [6], [7]

One of the earliest signs of CVD in individuals with CKD is endothelial damage and dysfunction, [8] and this has been shown even in children with pre-dialysis CKD. [9] In this context, potential causes of endothelial damage and aberrant repair are disturbances in growth factors involved in the formation of vascular networks. [10] Angiopoietin-1 (Ang-1) binds and activates the Tie-2 receptor on endothelia where it promotes cell survival and decreases vascular permeability. [11] As such, Ang-1 is usually considered beneficial for endothelial cell function. In contrast, Ang-2 is released from Weibel-Palade bodies by various stimuli [12], [13] and acts as an antagonist of Ang-1. [14] Ang-2 has pro-inflammatory actions [15], [16] and can also promote or retard angiogenesis dependent on the ambient levels of vascular endothelial growth factor-A (VEGF-A). [14] Other evidence exists that, in certain circumstances, Ang-2 may have biological effects, independent of the antagonism of Ang-1. [17], [18]

Elevated circulating Ang-2 has been reported in adults with CKD. David and colleagues [19] found an inverse relationship between circulating Ang-2 levels and glomerular filtration rate in adults with CKD. Two other studies reported that Ang-2 levels were elevated in adults on hemodialysis (HD) or peritoneal dialysis (PD) compared with healthy controls. [20], [21] In one of these studies Ang-2 correlated with scoring for coronary and peripheral arterial disease. [20] In the other study, Ang-2 correlated with cholesterol, high-sensitive C-reactive protein and osteoprotegerin and was an independent predictor of mortality. [21]

To date, no clinical studies have examined angiopoietins in childhood CKD, despite the latter having similar cardiovascular complications as adults with CKD, but at a proportionately earlier age. [1] These effects are more likely to be directly attributed to the uremic milieu because children seldom have diabetes or dyslipidaemia, uncontrolled hypertension or are smokers which themselves predispose to CVD. We hypothesized that an imbalance of angiopoietin vascular growth factors, which would be detrimental to endothelial structure and function, might be present in children with CKD. Specifically, we predicted that childhood CKD would be associated with elevated Ang-2 and that it would correlate with inflammatory markers.

## Materials and Methods

### Patient cohort

Informed written consent was obtained from the next of kin, caretakers, or guardians on the behalf of the minors/children participants, and children also gave their assent where appropriate. The study was approved by the Great Ormond Street Hospital and UCL Institute of Child Health research ethics committee. From January to December 2010, 20 children in pre-dialysis CKD stages 4-5 and 30 on dialysis (14 PD, 16 on HD) were recruited from Great Ormond Street Hospital. Primary diagnoses included renal dysplasia (n = 20), posterior urethral valves (n = 9), focal segmental glomerulosclerosis (n = 6), nephronophthisis (n = 4), cortical necrosis (n = 3), and 2 each with autosomal recessive polycystic kidney disease, congenital nephrotic syndrome, bilateral Wilms’ tumors and unknown causes. None of the children had diabetes and none were smokers. Children with underlying inflammatory disorders, such as glomerulonephritis and vasculitides were excluded. Patients were compared with healthy age- and gender- matched children who formed part of a contemporaneous study and are previously described. [22]

### Clinical, biochemical and vascular parameters

All measures were taken at the same clinical visit; pre a mid-week session of HD or at clinic review for pre-dialysis CKD and PD patients. All children had their weight, height, body mass index (BMI) and Doppler blood pressure measured; these were expressed as standard deviation score (SDS) for age and gender. [23] Routine blood tests (including creatinine, calcium, ionized calcium, phosphate, parathyroid hormone and serum urate) were performed. All children above 5 years of age (n  =  24 children on dialysis [11 on PD, 13 on HD]; 14 children in pre-dialysis CKD and 25 healthy controls) underwent vascular scans to assess carotid artery intima media thickness (cIMT) and aortic pulse wave velocity (PWV) using methods previously described [24] and expressed as SDS for age. [25], [26] Serum was obtained and ELISA used to assess circulating levels of human Ang-1, Ang-2, VEGF-A, Flt-1, E-selectin, P-selectin, intracellular adhesion molecule 1 (ICAM-1) and vascular cell adhesion molecule 1 (VCAM-1) (R & D Systems). In some cases, serum samples were taken both pre- and post- HD.

### Immunolocalisation of Ang-1, Ang-2 and VEGF-A in intact arteries

Medium-sized muscular arteries routinely removed at omentectomy during a peritoneal dialysis catheter insertion or at renal transplantation were obtained from some of the pre-dialysis CKD and dialysis patients (n = 4 in each group). [3] Tissues were fixed in formalin, embedded, then sections cut for immunohistochemistry as described [27] for the following antibodies: rabbit anti-mouse Ang-1 (ADI); rabbit anti-mouse Ang-2 (ADI); rabbit anti-human VEGF-A (Santa Cruz) and rabbit anti-human von Willebrand factor (DAKO). Intensity of staining was quantified by a blinded observed and scored between 0 (no reactivity) to 3 (strong staining); at least four images were obtained from each vessel and a mean value obtained for each specimen.

### Uric acid stimulation of human umbilical vein endothelial (HUVEC) and aortic smooth muscle cells (HAoSMC)

HUVEC and HAoSMC (Lonza) were cultured in either EGM-MV or DMEM supplemented with 20% FBS, 25 mM HEPES, 100 U/ml penicillin and 100 mg/ml streptomycin respectively. Cells from passage 2–4 were grown to 70% confluence, placed in low-serum media for 24 hours and challenged with varying concentrations of uric acid (3–12 mg/dl) [28] for 15 minutes, 24 hours and 72 hours. Conditioned media was collected at all time-points to assess Ang-2 levels and cell lysates extracted for protein measurements. In other experiments, RNA was extracted from cells stimulated with uric acid for 6 hours and used for RT-PCR for *Ang1, Ang2,* organic anion transporters 1–4 (*Oat1-4*) *Tie1, Tie2,* Toll-like receptor 4 *(Tlr4)* and human uric acid transporter 1 (*Urat1*) using previously described methods. [28] Quantitative RT-PCR was also performed for *Ang2* on HUVEC exposed to uric acid (n = 3 for each dose) with hypoxanthine-guanine phosphoribosyltransferase (HPRT) used as a house-keeping gene. Primer details available on request.

#### Statistics

Results are presented as mean ± SD or median and inter quartile range (IQR), depending on the distribution. Univariate comparisons of continuous variables were performed using unpaired *t*-test for normally distributed data, or non-parametric Mann-Whitney U-test for non-normally distributed variables. For multiple comparisons of several groups, ANOVA or Kruskall-Wallis test were performed. Within group comparisons of continuous variables were performed using paired *t*-test or Wilcoxon test, as appropriate. Spearman tests were used for correlation analyses. Interactions between Ang-2 and biochemical data or vascular scans were tested by two way ANOVA and the difference between each pair of means compared by Tukey’s test with appropriate adjustment for the multiple testing. Factors affecting the two outcome variables, Ang-2 and cIMT, were explored using multiple regression analysis, including all variables with *p* ≤0.15 from univariate analysis in the stepwise multiple regression models. For all analyses, *p* <0.05 was considered statistically significant.

## Results

### Circulating Ang levels in pre-dialysis CKD and dialysis patients

Demographic and clinical parameters of the groups studied are summarized in **Table 1**. The pre-dialysis CKD and dialysis patients were similar in all demographic, clinical and biochemical markers except that 25-hydroxyvitamin D was lower and serum cholesterol and urinary albumin/creatinine ratio higher in dialysis patients (**Table 1**). The healthy controls had significantly higher BMI SDS and lower blood pressure SDS and urate levels *versus* the patients.

**Table 1:** Demographic, clinical, anthropometric, and biochemical characteristics of patients and control subjects.

Characteristics	Pre-dialysis CKD (n = 20)	Dialysis (n = 30)	Healthy Controls (n = 25)	p
Age (yr)	10.7±4.1	14.2±3.9	13.1±2.8	0.68
Sex (males/females)	12/8	17/13	14/11	0.82
Race (White/Asian/Black/other)	14/5/1/0	19/7/2/2	16/7/2/0	0.85
eGFR (ml/min per 1.73 m^2^)	18.3±6.0	-	113±9.8	-
Time in CKD4-5 pre-dialysis (yr; median [IQR])	4.5 (1.1–9.2)	3.9 (0.2–7.9)	-	0.70
Time on dialysis (yr; median [IQR])	-	1.4 (0.2–3.9)	-	-
Dialysis modality (PD/HD)	-	14/16	-	-
BMI SDS	−0.6±1.1	−0.7±0.3	1.1±0.7	0.42
Systolic BP index*	1.9±0.8	1.5±2.5	0.8±0.2	0.21
Numbers on antihypertensive medications	11	4	0	0.6
Numbers on ACEi or ARB	4	0	0	0.1
Hemoglobin (g/dl)	12.3±1.9	11.4±0.8	12.1±0.9	0.51
Albumin (g/L)	39.0±4.1	41±4.8	40±0.6	0.34
Total cholesterol (mmol/L)	3.5±1.3	4.1±0.9	3.1±0.7	0.07
Triglycerides (mmol/L)	1.1±0.7	1.4±2.1	0.9±0.6	0.11
No. on statins	0	1	0	-
Albumin-adjusted calcium (mmol/L)	2.4±0.2	2.4±0.1	2.4±0.2	0.9
Serum phosphate levels (mmol/L)	1.4±0.6	1.6±0.8	1.2±0.2	0.9
Parathyroid hormone (pmol/L)	5.2±1.1	8.9±3.7	-	0.06
Serum urate level ( µmol/L)	260±20.8	278±29.3	184±33.0	0.88
25-hydroxyvitamin D (nmol/L)	40.1±16.2	12.9±9.8	-	0.04
Urinary albumin / creatinine ratio (mg/mmol)	122.8±18.6	260.0±64.3 (n = 21)	-	0.04

All values are presented as mean±SD; p value indicates comparisons between the pre-dialysis CKD and dialysis groups. Parathyroid hormone, 25-hydroxyvitamin D and urinary albumin / creatinine ratio were not measured in healthy controls due to small volumes of serum and lack of urine samples. * Systolic BP index  =  measured BP/95th centile BP for age, gender, and height. ARB, angiotensin II receptor blocker; ACEi, angiotensin-converting enzyme inhibitor; BMI, body mass index; SBP, systolic BP; SDS, SD score.

**Figure 1:**
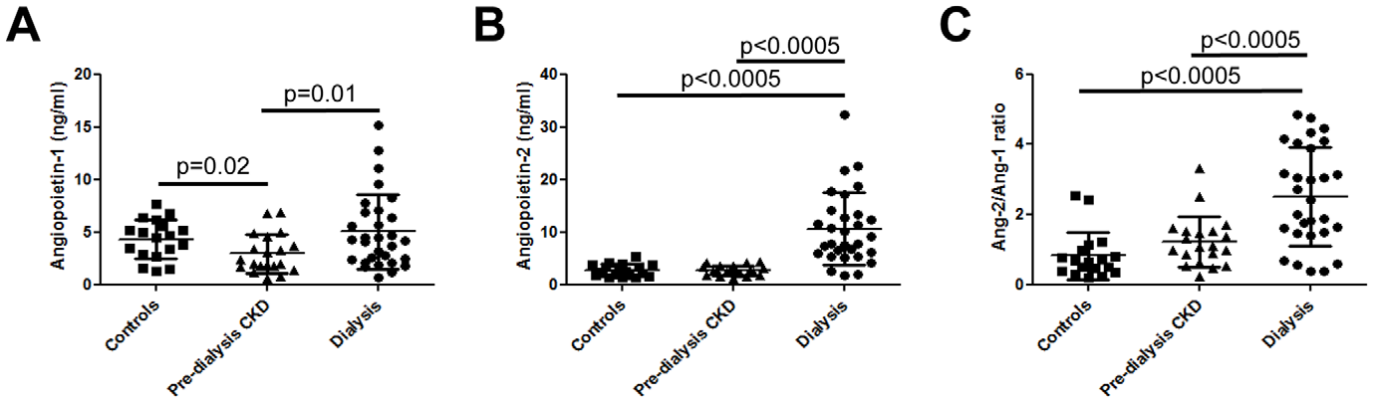
Circulating Ang levels in pre-dialysis CKD and dialysis patients. Serum Ang-1 levels (A) were significantly lower in pre-dialysis CKD patients compared with healthy controls. In dialysis patients Ang-1 levels were similar to values found in healthy controls. Similar levels of both circulating Ang-2 (B) and Ang-2/Ang-1 (C) were found in healthy children and those with pre-dialysis CKD, but these were significantly increased in the dialysis group.

Ang-1 levels were modestly but significantly (p = 0.02) lower in pre-dialysis CKD patients compared with healthy controls (respective means±SD being 2.9±1.8 and 4.3±1.8 ng/ml). In dialysis patients, Ang-1 levels (mean±SD 5.0±3.5 ng/ml) were similar to values found in healthy controls. Circulating Ang-2 levels were not significantly different between healthy children and those with pre-dialysis CKD, but were markedly and significantly increased in the dialysis group (means±SD in controls 2.7±1.2, pre-dialysis CKD 2.7±0.9, dialysis 10.5±6.9 ng/ml, p<0.0005 in comparisons between dialysis patients and both the other groups). As explained in the *Introduction*, Ang-2 acts an endogenous antagonist to Ang-1, such that comparative levels may be relevant; hence we evaluated the Ang-2/Ang-1 ratio. There was no difference in the Ang-2/Ang-1 ratios (**Figure 1C**) between control and pre-dialysis CKD individuals but it was significantly higher in dialysis patients compared with the other groups (means±SD in controls 0.8±0.7, pre-dialysis CKD patients 1.2±0.7 and dialysis patients 2.5±1.4 ng/ml, p<0.0005 in comparisons between dialysis and both the other groups)

### Correlation of Ang levels with clinical and vascular measures

**Figure 2:**
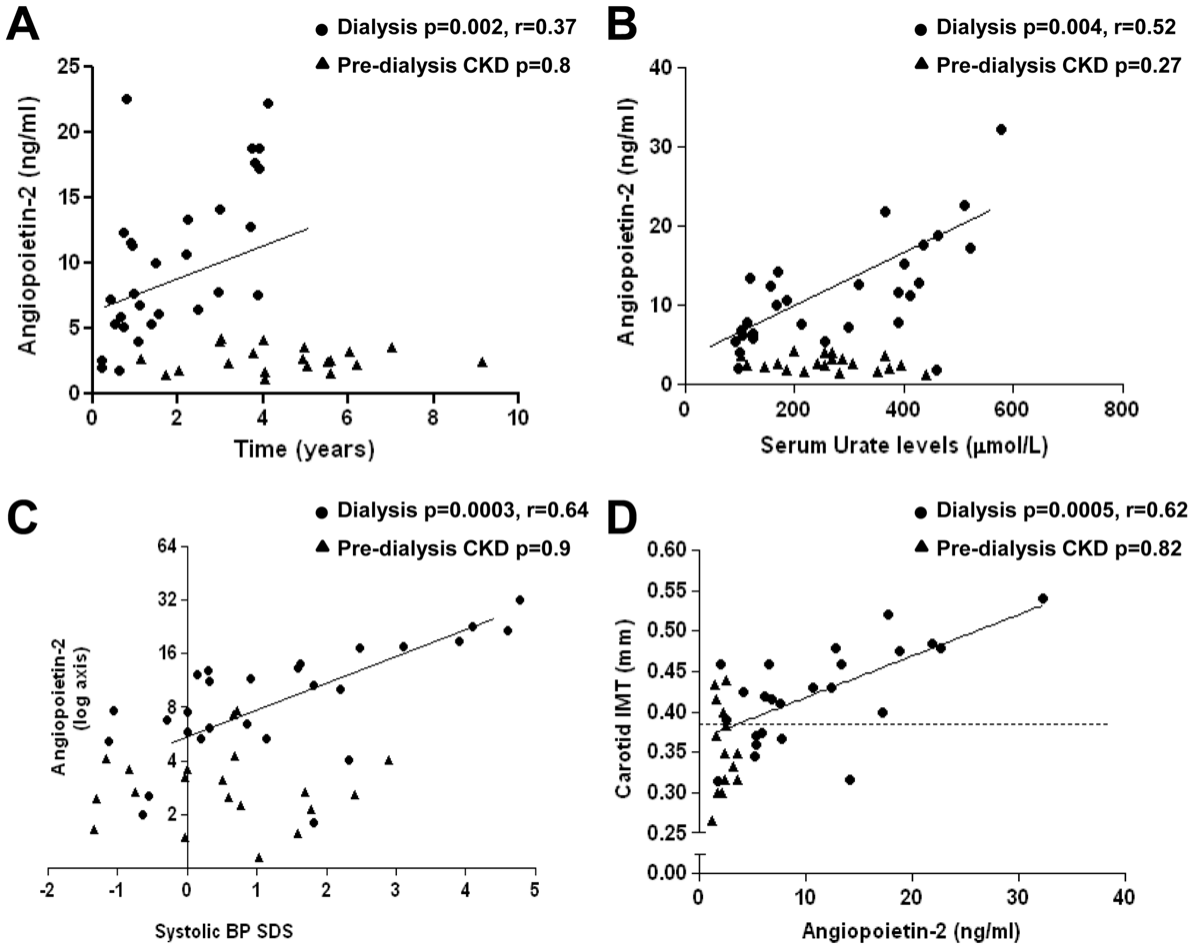
Correlation of Ang-2 levels with clinical and vascular parameters. Ang-2 levels in dialysis individuals correlated positively with time on dialysis (A), serum urate levels (B), systolic blood pressure SDS (C) and cIMT (D). Independent variables are shown on the x-axis. Regression lines account for dialysis patients only. Dotted line in D indicates the value for cIMT in healthy age-matched controls. There was no correlation between Ang-2 and any clinical and vascular measures in pre-dialysis CKD patients.

Ang-2 levels had no significant relation to age or gender, but increased linearly with time on dialysis (r = 0.37, p = 0.002) whereas there was no association of Ang-2 levels with the time spent in pre-dialysis CKD (p = 0.8, **Figure 2A**). There was no difference in Ang-2 levels between HD and PD patients. Circulating Ang-2 levels was also not significantly related to the presence of residual renal function. To determine whether Ang-2 was cleared by HD, we obtained serum samples pre- and post-HD from 5 individuals. There was no significant differences in Ang-2 levels (means±SD 5.0±1.1 and 4.6±0.9 ng/ml, p = 0.7).

Serum urate levels were significantly increased in both pre-dialysis CKD and dialysis patients compared with controls (**Table 1**) and showed a weak positive correlation with systolic blood pressure SDS in these patients (r = 0.12, p = 0.048). Urate levels positively correlated with Ang-2 levels in the dialysis group (r = 0.52, p = 0.004, **Figure 2B**). There was a strong positive correlation between Ang-2 levels and systolic blood pressure SDS in the dialysis patients (r = 0.64, p = 0.003), but not in the pre-dialysis CKD group, **Figure 2C**). No significant correlations were found between Ang-1 or Ang-2/Ang-1 ratio with any clinical, biochemical or vascular parameters.

Three out of 14 (21%) pre-dialysis CKD patients had increased cIMT compared with age-matched controls (0.37±0.03 versus 0.38±0.02 mm respectively), but there was no significant correlation between Ang-2 and cIMT in this group (p = 0.82, **Figure 2D**). In contrast, cIMT was increased in 16 of 24 (66%) dialysis patients (0.46±0.05 mm) and showed a strong positive correlation with Ang-2 (r = 0.62, p = 0.0005, **Figure 2D**). PWV was increased in two out of 14 children with pre-dialysis CKD (5.1±0.2 m/sec in pre-dialysis CKD versus 5.0±0.3 m/sec in controls) and 7 out of 24 (5.6±0.5 m/sec) on dialysis but did not show any correlation with Ang-2 in either group. Ang-2 levels were not significantly correlated with blood cholesterol, triglyceride, albumin, calcium, phosphate, parathyroid hormone, 25-hydroxyvitamin D or urinary albumin/creatinine levels in pre-dialysis CKD or dialysis patients. On multiple regression analysis the significant determinants of Ang-2 levels were systolic blood pressure and serum urate levels (**Table 2**). Carotid IMT was significantly and independently influenced by the time on dialysis, calcium x phosphate product and Ang-2 levels (**Table 2** **)**.

**Table 2:** Multiple regression analyses for independent predictors of Angiopoietin-2 (Ang2) and carotid intima media thickness (cIMT).

Variables	β	SE	p	Model R^2^
*Ang2*				71%
*Systolic BP*	2.54	0.21	<0.001	
*Serum urate level*	0.14	0.006	0.03	
*cIMT*				68%
*Time on dialysis*	0.50	0.02	0.008	
*Ca x P product*	0.37	0.12	0.02	
*Ang2*	0.26	0.06	0.05	

β - Unstandardized regression coefficient; indicates the difference in the outcome variable (Ang2 or cIMT) per unit change in the independent variables.

SE – standard error

Model R^2^ - The amount of variance in the dependent variable that can be explained by the model.

### Circulating levels of VEGF-A and sFlt-1

**Figure 3:**
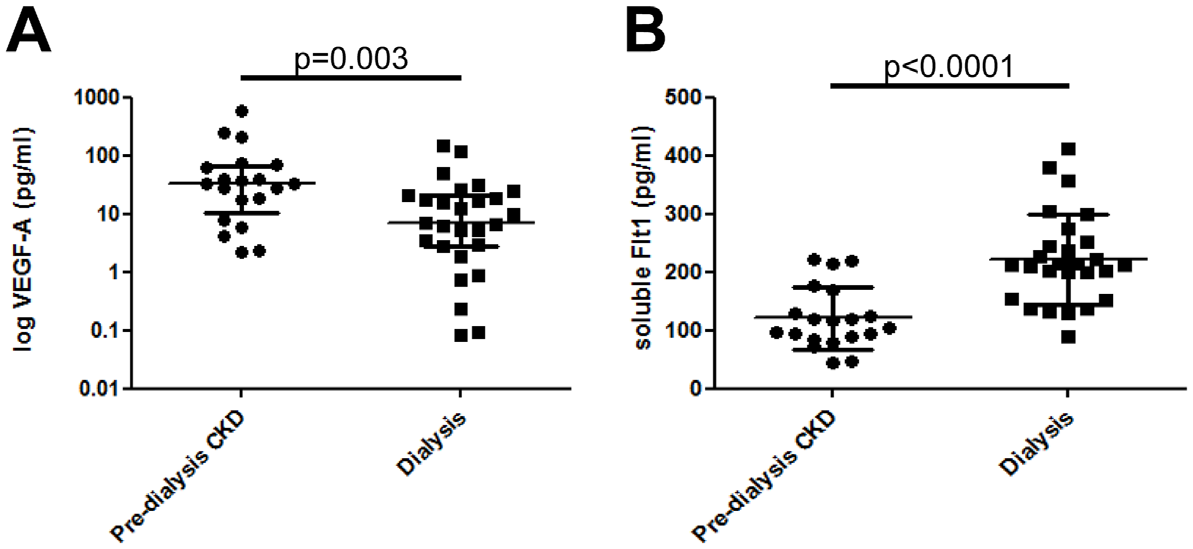
Circulating levels of VEGF-A and sFlt-1 in pre-dialysis CKD and dialysis patients. VEGF-A levels were significantly lower in individuals on dialysis compared with pre-dialysis CKD patients (A). In contrast, sFlt-1 were significantly higher in the dialysis patients (B)

The biological actions of Ang-2 on blood vessels are dependent on VEGF-A availability; [14] so we measured circulating levels of this growth factor and the endogenous VEGF-A inhibitor, sFlt-1. Ang-2 levels were similar in healthy controls and pre-dialysis CKD patients and therefore VEGF-A and sFlt-1 levels were only measured in pre-dialysis CKD and dialysis patients. VEGF-A levels were significantly lower in individuals on dialysis compared with pre-dialysis CKD patients (respective medians being 6.9 and 33.5 pg/ml, p = 0.003, **Figure 3A**). In contrast, sFlt-1 levels were significantly higher in dialysis patients compared with pre-dialysis CKD (respective means±SD of 222±78 and 121±54 pg/ml respectively, p<0.0001, **Figure 3B**). There were no significant correlations of either VEGF-A or sFlt-1with time or mode of dialysis, urate levels, blood pressure SDS or any other vascular measures.

### Circulating levels of soluble cell adhesion molecules

**Figure 4:**
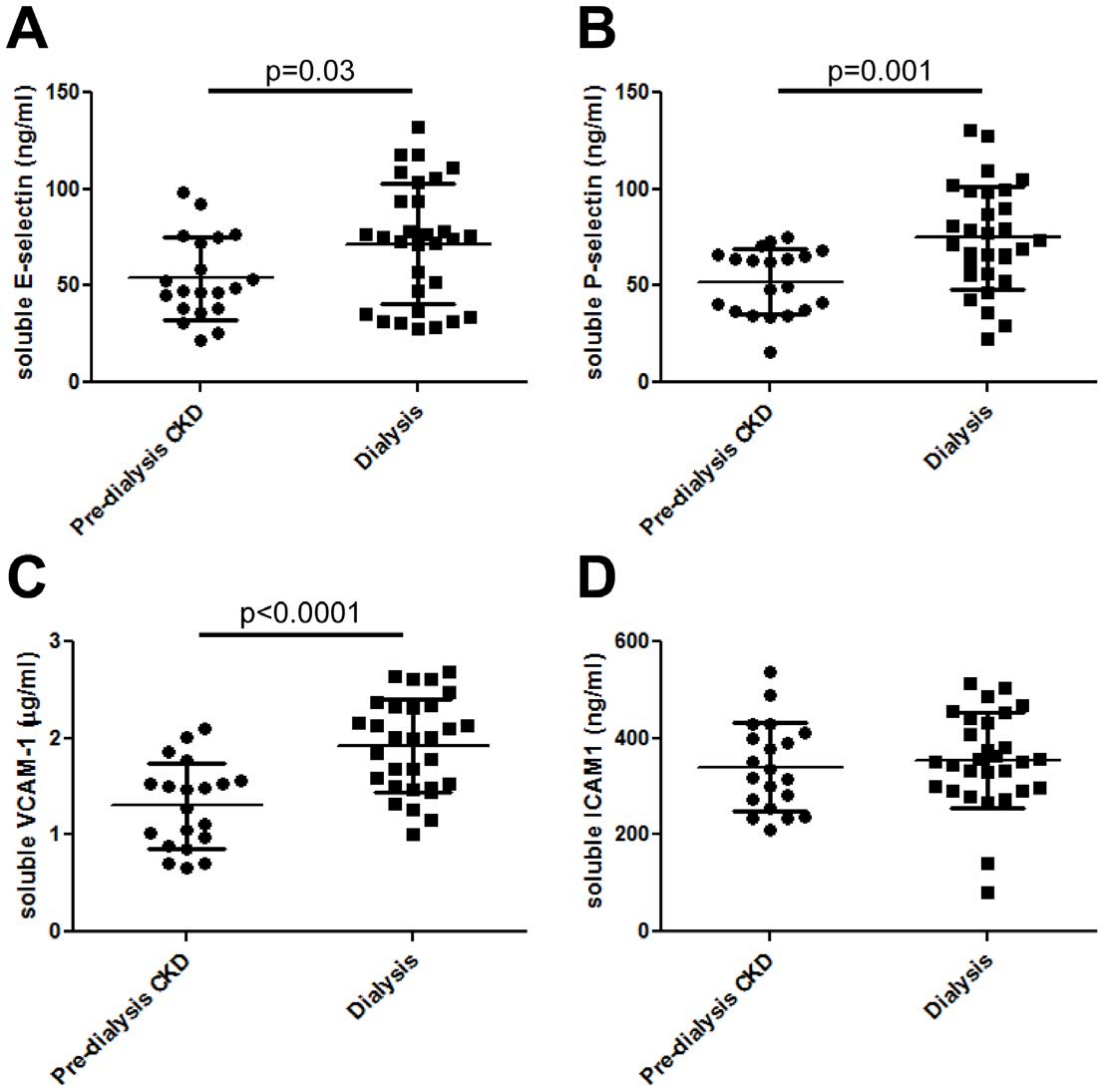
Circulating levels of soluble cell adhesion molecules. Compared with pre-dialysis CKD individuals, patients treated with dialysis had significantly elevated levels of soluble E-selectin (A), P-selectin (B) and VCAM-1 (C); there was no difference in ICAM-1 levels (D).

Ang-2 has been shown to have pro-inflammatory actions. [16], [17] Therefore, circulating levels of cell adhesion molecules which attract inflammatory cells were measured. [29] Compared with pre-dialysis CKD individuals, patients treated with dialysis had significantly elevated levels of soluble E-selectin (respective means±SD being 72±31 and 54±21 ng/ml, p = 0.03, **Figure 4A**), soluble P-selectin (75±27 *versus* 52±17 ng/ml, p = 0.001, **Figure 4B**) and soluble VCAM-1 (1.9±0.5 *versus* 1.3±0.4 µg/ml, p<0.0001, **Figure 4C**) but there was no difference in ICAM-1 (354±99 versus 341±91 ng/ml, **Figure 4D**). In the dialysis population, circulating levels of Ang-2 positively correlated with soluble VCAM-1 (r = 0.41, p = 0.02), but there were no significant correlations with E-selectin, ICAM-1 or P-selectin.

### Immunolocalisation of vascular growth factors in arteries

**Figure 5:**
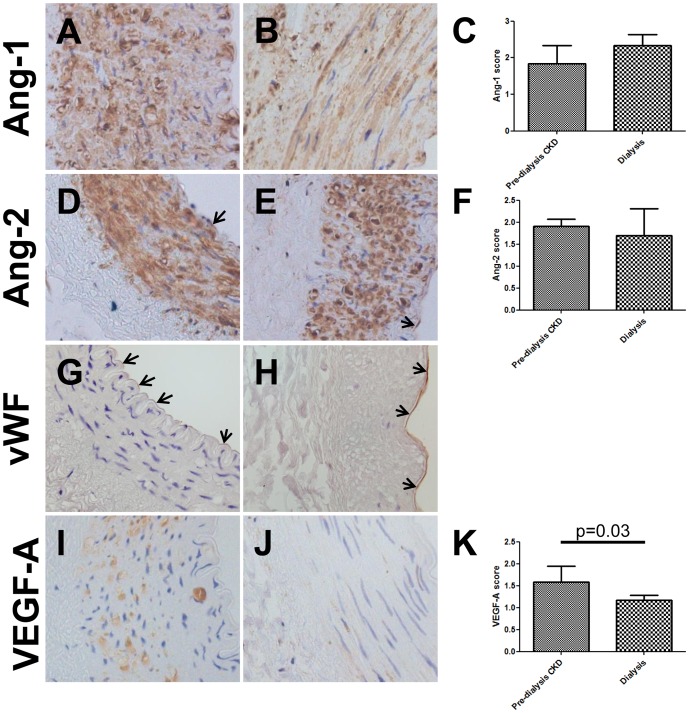
Immunolocalisation of vascular growth factors in arteries. Ang-1 was detected in the media of vessels from both pre-dialysis CKD (A) and dialysis patients (B); no differences in staining intensity were observed between the two groups (C). Ang-2 was immunodetected in both the media and endothelia (arrows) in pre-dialysis CKD (D) and dialysis (E) vessels with similar intensity (F). The endothelial later was also positive for von Willebrand factor (arrows, G and H). VEGF-A immunostaining was prominent in the media of pre-dialysis CKD vessels (I), but was significantly decreased in dialysis patients (J and K). All fields taken with ×40 objective.

To seek potential source(s) of Ang-1, Ang-2 and VEGF-A immunohistochemistry was undertaken on intact arteries obtained from a subset of the pre-dialysis CKD and dialysis patients. [3] Ang-1 protein was detected in the media of vessels from pre-dialysis CKD (**Figure 5A**) and dialysis patients (**Figure 5B**). As scored by an observer blinded to the source of the samples, there was no difference in staining intensity between the two groups (**Figure 5C**). Ang-2 was also immunodetected in the media of both pre-dialysis CKD (**Figure 5D**) and dialysis (**Figure 5E**) vessels with a similar intensity in each group (**Figure 5F**). Ang-2 expression was also detected in the endothelial layer which was also positive for von Willebrand factor (**Figure 5G, 5H**). VEGF-A immunostaining was prominent in the media of pre-dialysis CKD vessels (**Figure 5I**), but was significantly decreased in dialysis patients (**Figure 5J and K**).

### Effect of uric acid exposure on Ang-2 release in-vitro

**Figure 6:**
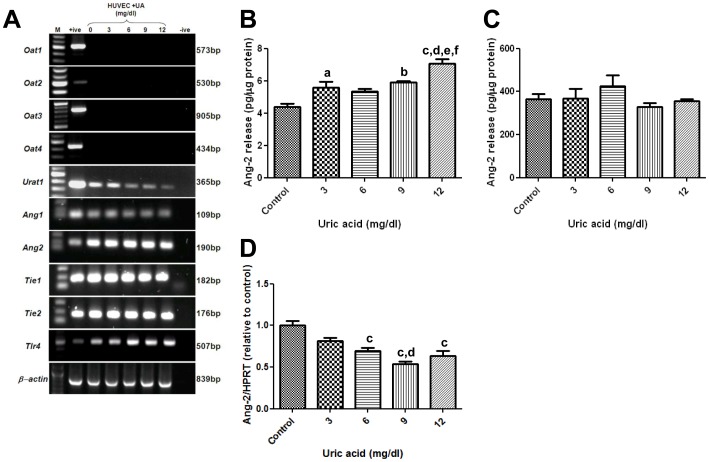
Effect of uric acid on Ang-2 secretion in HUVECs. A) Both non-stimulated and uric acid stimulated HUVECs expressed the mRNA for the transporter *Urat1* but not *Oat1-4*; they were also positive for *Ang-1*, *Ang-2*, *Tie-1*, *Tie-2 and Tlr4*. Sizes were determined using a 100 bp marker (m), positive (+ive) controls consisted of total kidney cDNA and negative controls were without cDNA addition. (B) Uric acid stimulation for 15 minutes, but not 72 hours (C) led to elevated Ang-2 secretion in the conditioned media of HUVEC cells. Within the cells, uric acid stimulation led to a decreased abundance of Ang-2 mRNA after 6 hours of stimulation (D). a = p<0.05 compared with controls, b = p<0.01 compared with controls, c = p<0.001 compared with controls, d = p<0.01 compared with HUVEC stimulated with 3 mg/dl uric acid, e = p<0.01 compared with HUVEC stimulated with 6 mg/dl uric acid, f = p<0.05 compared with HUVEC stimulated with 9 mg/dl uric acid.

As demonstrated above, Ang-2 levels strongly and positively correlated with urate levels in dialysis patients. We hypothesized that elevated urate might increase Ang-2 expression by, and/or release from, endothelial and/or vascular smooth muscle cells. There has been a previous report that uric acid can stimulate release of contents from Weibel-Palade bodies including Ang-2. [13] We first examined how urate may enter HUVEC and detected the urate transporter, *Urat1*, but not *Oat1-4*; the mRNA levels of *Urat1* showed a tendency to decrease with increasing doses of uric acid stimulation (**Figure 6A**). HUVEC also expressed *Ang-1*, *Ang-2*, *Tie-1* and *Tie-2* (**Figure 6A**).

Exposure of HUVECs to uric acid for 15 minutes led to an increase in Ang-2 release *versus* control media as evaluated by the proportion of Ang-2 protein in the conditioned media. The most prominent response was observed with 12 mg/dl with Ang-2 levels significantly elevated compared with all other groups (**Figure 6B**). This was an acute effect because longer term stimulation with uric acid for 72 hours did not enhance the release of Ang-2 protein in the conditioned media (**Figure 6C**). Within the cells, uric acid stimulation led to a decreased abundance of *Ang-2* mRNA after 6 hours of stimulation (**Figure 6D**), compared with controls. It has been suggested that the acute release of Ang-2 from endothelia is mediated by *Tlr4* [13] and we detected mRNA levels of *Tlr4* on HUVECs (**Figure 6A**). Prior studies have shown that HAoSMC express the *Urat1* receptor [28] and in the current study they were also found to express transcripts for *Ang-1*, *Ang-2* and *Tie-2*, but not *Tie-1* (data not shown); however, we did not detect Ang-2 protein in the conditioned media with/without addition of uric acid.

## Discussion

Our study demonstrated that circulating Ang-2 levels were markedly elevated in dialysis patients compared with healthy controls and pre-dialysis CKD individuals. Amongst the dialysis patients, Ang-2 positively correlated with time on dialysis, systolic blood pressure and cIMT, but not PWV. These findings may indicate that circulating Ang-2 is a marker for the early cardiovascular changes occurring in children with CKD on dialysis. Previous studies have demonstrated that in the more compliant vessels of children with CKD structural changes precede functional alterations with increases in cIMT observed before alterations in PWV. [30] Furthermore, our work examining intact vessels from children on dialysis indicated that the vessel calcium load showed a strong linear association with cIMT but not with PWV or the coronary calcification score. [3]

Our findings concur with several studies that have shown a relationship between circulating Ang-2 levels and cardiovascular complications in adults. Elevated circulating Ang-2 is associated with scores for coronary and peripheral arterial disease in adults with CKD on PD or HD [20] and positively correlated with systolic blood pressure and left ventricular hypertrophy in 4000 young to middle-aged individuals. [31]. A further study [21] demonstrated that Ang-2 was an independent predictor of mortality in CKD patients and correlated with markers of vascular disease (cholesterol, hsCRP and osteoprotegerin) but not the degree of vascular calcification or arterial stiffness. The observation that circulating Ang-2 is also elevated in children on dialysis suggests that the uraemic environment may directly influence vascular growth factor expression. This is because children do not have many of the cardiovascular comorbidities that are commonly seen in adults. In addition, the pathophysiology of CVD in children may be different to that found in adults, for example, our previous work has shown that children on dialysis develop arteriosclerosis with exclusively medial involvement [3] whereas adults are much more likely to have both intimal lesions as well as medial damage [32]. Therefore results from adults may not be able to be directly extrapolated to the paediatric population and studies in children with CKD are necessary.

Our studies found that the elevation in circulating Ang-2 levels were similar immediately before and after a HD session. Both Ang-1 and Ang-2 form multimeric structures composed of monomers of 55 kDa [33] and therefore unlikely to be affected by dialytic clearance. In contrast to adults with CKD [19], [21] we did not detect different Ang-2 levels in children with pre-dialysis CKD compared with healthy controls. One explanation for this discrepancy could be that the children under study had not been exposed to diabetes mellitus, and that dyslipidaemia and hypertension were less common than in adults with CKD. Indeed, each of these factors have been shown to be associated with elevated Ang-2. [31], [34] Instead, children with pre-dialysis CKD had decreased circulating Ang-1 compared with healthy controls. This loss of Ang-1 in pre-dialysis CKD children may decrease blood vessels stability and be an early sign of the endothelial dysfunction which occurs in these patients. [9] Potential sources of Ang-1 not only include the vessel wall, but also platelets [35] and one caveat to consider when measuring circulating Ang-1 in serum samples is that ex-vivo activation may increase Ang-1 levels within serum tubes. [36] In future studies, it would be of interest to quantify both circulating Ang-1 and platelet-derived Ang-1.

Elevated circulating Ang-2 levels in dialysis *versus* non-dialysis CKD children were associated with an anti-angiogenic environment as demonstrated by decreased circulating VEGF-A and elevated soluble sFlt-1 (**Table 3**). Increased sFlt-1 [37] and reduced circulating VEGF-A [38] have been demonstrated in adult populations with CKD. In the presence of low VEGF-A, Ang-2 will destabilise blood vessels leading to vessel regression. [14] This milieu of growth factors may therefore contribute to the impaired endothelial function seen in CKD children on dialysis. [9], [39]

**Table 3:** Changes in circulating angiogenic and inflammatory markers between pre-dialysis CKD and dialysis patients.

Circulating marker	Levels in dialysis compared with pre-dialysis CKD patients
*Angiogenic factors*	
Ang-1	No change
Ang-2	↑
Ang-2/Ang-1 ratio	↑
VEGF-A	↓
sFlt-1	↑
*Inflammatory markers*	
E-selectin	↑
P-selectin	↑
VCAM-1	↑
ICAM-1	No change

Increased circulating Ang-2 in CKD children on dialysis was also associated with pro-inflammatory responses with high urate, E-selectin, P-selectin and VCAM-1 (**Table 3**). Systemic inflammation is seen in children with CKD with dialysis [3], [40] and Ang-2 may play a direct role in this process. Ang-2 can sensitise the endothelium to inflammatory responses; [16] and directly affect the biology of inflammatory cells which express the Tie-2 receptor themselves. [41], [42] Although, we demonstrated that Ang-2/Ang-1 was also elevated in CKD children on dialysis it did not correlate with any cardiovascular parameters. This suggests that the total Ang-2 levels are important in biological responses in dialysis patients, rather than the relative balance between Ang-2 and Ang-1.

We detected Ang-2 in the endothelium of intact arteries isolated from children with CKD and cultured HUVECs indicating this cell type is a potential source of the increased Ang-2 in dialysis patients. Our studies detected Ang-2 in the walls of intact arteries from both pre-dialysis CKD and dialysis patients and *Ang-2* transcripts were detected in cultured HoASMCs. Using *Ang-2/LacZ* mice positive expression in renal arterial walls during kidney development has been observed; [43] whilst Ang-2 has been detected in cultured mouse embryonic fibroblasts [44] and smooth muscle cells derived from the heart microvasculature. [45] We could not detect any Ang-2 released from HoASMCs suggesting vascular smooth muscle cells may not contribute to the increase in Ang-2 seen in dialysis patients. We cannot rule this out completely as the cells used in these experiments were not derived from patients, nor did we reproduce the uremic milieu they will be exposed to *in-vivo*. Another potential source of Ang-2 are macrophages. [41], [46] Although prior studies [3] have demonstrated that macrophages are not present in the intact arteries of children on dialysis they may be found in the circulation and increase Ang-2.

There are several potential mechanisms for the increase in circulating Ang-2 in patients with CKD. The increase in Ang-2 may be a direct consequence of elevated blood pressure. Korff and colleagues [47] demonstrated that hypertension in mice led to release of stored Ang-2 from Weibel-Palade bodies. There is also evidence that mediators of vascular tone such as angiotensin II can directly alter Ang-2 expression. [48] A lack of endothelial nitric oxide may also predispose to a release of Weibel-Palade bodies that would theoretically increase Ang-2 levels. [49]

One potential factor that could bring these various mechanisms together is uric acid. Urate is retained in CKD and found to correlate with Ang-2 levels in the dialysis patients. We showed that uric acid could directly induce the release of Ang-2 from HUVEC with a corresponding decrease in mRNA abundance within the cell, consistent with prior reports that uric acid stimulates release of Weibel-Palade bodies. [13] These effects are likely to be mediated by *Urat1* and *Tlr4* [13], both of which were found to be expressed on endothelia. Future studies using inhibitors specific for *Urat1* (probenecid [28] and *Tlr4* (TAK-242 [50] would help to determine the specific role of these molecules in Ang-2 release from endothelia exposed to uric acid. In addition, there is increasing evidence that urate may have a role in hypertension via effects that include inducing endothelial dysfunction, oxidative stress and the production of angiotensin II. [51] These findings might account for why urate can contribute to cardiovascular complications. [52], [53]

In conclusion, Ang-2 is a marker for cardiovascular disease in children on chronic dialysis. Furthermore, we suggest that Ang-2 may also be an anti-angiogenic and pro-inflammatory effector in this context.
